# Rapid identification of growth years and profiling of bioactive ingredients in *Astragalus membranaceus* var. mongholicus (*Huangqi*) roots from Hunyuan, Shanxi

**DOI:** 10.1186/s13020-017-0135-z

**Published:** 2017-05-19

**Authors:** Hua-Sheng Peng, Jun Wang, He-Ting Zhang, Hai-Yan Duan, Xiao-Mei Xie, Ling Zhang, Ming-En Cheng, Dai-yin Peng

**Affiliations:** 1 School of Pharmacy, Anhui University of Chinese Medicine, Hefei, 230031 China; 2 State Key Laboratory Breeding Base of Dao-di Herbs, China Academy of Chinese Medical Sciences, Beijing, 100700 China; 3 School of Pharmacy, Bozhou Vocational and Technical College, Bozhou, 236800 China; 4 Institute of TCM Resources Protection and Development, Anhui Academy of Chinese Medicine, Hefei, 230031 People’s Republic of China

**Keywords:** Growth rings, Freehand section, Rotten heart, Histochemical localization, Concentration

## Abstract

**Background:**

The content of medicinal bioactive constituents in *huangqi* is affected by plant age. In this study, we devised a quick and convenient method for determining the age of *huangqi*, which was cultivated in Hunyuan County (Shanxi Province).

**Methods:**

1, 2, 3, 4, 5, 8, 10 growth years *huangqi* had 38 samples, all samples were collected separately. The growth rings in these samples were observed after making paraffin section and freehand-section. The relationship between growth rings and its growth years was analyzed by SPSS 19.0 software. Histochemical localization of total flavones and saponins in *huangqi* was determined by color reactions. The concentration of four flavonoids and two saponins in the roots of *huangqi* of different ages and different organizational structure (normal roots and rotten heart roots) were determined by HPLC-DAD and HPLC-ELSD. The results were analyzed by SPSS 19.0 software.

**Results:**

All *huangqi* samples had clear growth rings, and the statistical result about growth rings (X) and growth years (Y) showed significant correlation (r = 1, P = 0.000). The calibration curves of these six ingredients showed good linearity respectively, with significant correlation. All relative standard deviations (RSDs) of precision, recovery, repeatability, and stability experiments were less than 2%. Roots of 5-year-old plants contained the highest concentrations of total flavonoids and saponins. Saponin concentrations increased toward the center of the roots, whereas the four flavonoids showed an opposite trend in tissue distribution.

**Conclusion:**

The growth year of *huangqi* (Hunyuan County, Shanxi Province) could be determined soon and conveniently by naked eyes after staining phloroglucinol-HCl solution on freehand section. The content of saponins and flavonoids in rotten heart root and the surrounding normal tissues were affected by the formation and the extent of rotten heart.

**Electronic supplementary material:**

The online version of this article (doi:10.1186/s13020-017-0135-z) contains supplementary material, which is available to authorized users.

## Background

Astragali Radix, also known as *huangqi*, is a well-known Chinese herbal medicine. It is derived from the herbs *Astragalus membranaceus* var. *mongholicus* and *A. membranaceus* [1]. The main active compounds in *huangqi* are polysaccharides, saponins, flavonoids, and various trace elements [2–5]. *Huangqi* has immunoregulation properties, cardiovascular and cerebrovascular protection, and anticancer, antiviral, antiaging, and antidiabetic effects [6–12].

Geo-authentic medicinal materials (*Dao*-*di*) are produced in the natural conditions and ecological environment, e.g., the cultivation, harvesting, and processing techniques, leading to enhanced quality and clinical effects, compared with the original plant [13]. In the Qing dynasty, Shanxi Province became the *Dao*-*di* production area of *huangqi* [14], and Hunyuan County in Shanxi Province is currently one of the *Dao*-*di* production areas [15]. The accumulation of certain secondary metabolites in *huangqi* is related to the number of years of growth [16]. Consequently, the accurate and rapid determination of age is beneficial for evaluating the quality of *huangqi*. Growth rings have been discovered in the roots of many perennial herbs and have been used to identify plant age [17–22]. Growth rings have been found in *huangqi* and could be used to determine the age of samples [23].

The bioactive constituents of medicinal plants are often concentrated in particular tissues of the root [24, 25]. The quality evaluation standard of *huangqi* in Chinese pharmacopoeia is the contents of astragaloside A and calycosin-7-glucoside [1]. Flavonoid and saponin concentrations vary in the different tissue layers of *huangqi* [25, 26]. To date, however, there have been no studies that have investigated the influence of rotten heart on the distribution and concentration of bioactive constituents in *huangqi*.

In this study, we aimed to identify the age of *huangqi* cultivated in Hunyuan County (Shanxi Province) using a convenient method and to profile the main bioactive constituents. *Huangqi* of different ages were collected for anatomical examination and analysis of biochemical composition. This multidisciplinary approach enabled us to determine the affects of age, rotten heart, and tissue on the concentrations of the main active compounds.

## Methods

### Plant materials

Semi-wild *huangqi* of 1, 2, 3, 4, 5, 8, and 10 years of age was collected separately from Hunyuan County (Table 1) in September 2012. 1-year-old *huangqi* had 5 samples; 2-year-old *huangqi* had 8 samples; 3-year-old *huangqi* had 8 samples; 4-year-old *huangqi* had 8 samples; 5-year-old *huangqi* had 5 samples; 8-year-old *huangqi* had 3 samples and 10-year-old *huangqi* had 1 sample. The above-ground parts were retained as plant specimens. The roots were dried in the sun for experimental analysis. The roots of 8-year-old *huangqi* were categorized into normal roots and rotten heart roots. All samples were authenticated by Professor Huasheng Peng (School of Pharmacy, Anhui University of Chinese Medicine) with reference to the Flora of China [27].

**Table 1:** Sample information and number of growth rings observed using two different methods

Age (years of growth)	Number of specimen	TRR	TRM
1	5	0	1
2	8	1	2
3	8	2	3
4	8	3	4
5	5	4	5
8	3	^a^	8
10	1	^a^	10

*TRR* the number of growth rings observed with the naked eye after staining of transverse sections with phloroglucinol–HCl reagent, *TRM* the number of growth rings observed using light microscopy

^a^The number of growth rings was not clearly observed after staining of transverse sections with phloroglucinol–HCl reagent

### Chemicals and reagents

The internal standards calycosin-7-glucoside and astragaloside A were purchased from the National Institutes for Food and Drug Control (Beijing, China). Calycosin, formononetin, and astragaloside II were purchased from the Traditional Chinese Medicine Standardization Research Center (Shanghai, China). Ononin was purchased from Vic Biological Technology (Sichuan, China). Acetonitrile (TEDIA, USA) was chromatographically pure and the water was ultrapure from Milli-Q (Merck Millipore, USA). All other reagents were of analytical grade.

### Transverse section analysis

Segments of the roots of 10-year-old *huangqi* (length 80 cm, diameter 2.9 cm) were cut transversely (5 mm thick) at 5-cm intervals from the root-head to the root-tail. These segments were used to prepare paraffin sections. The paraffin sections were prepared as follows: initially, the segments were placed in FAA solution (70% ethanol:formaldehyde:acetic acid, 90:5:5) for over 24 h. The segments were then dehydrated using a gradient series of alcohol, and thereafter embedded in paraffin. Subsequently, the embedded roots were serially sectioned at 10–15-μm thickness using a Leica RM2265 rotary microtome. The serially sectioned samples were placed on clear slides in a consecutive order and then baked for more than 24 h. Each section was then deparaffinized and stained with safranin-fast green and safranin reagent. The parenchyma stained green and the vascular tissue stained red. The microscopic structure of different parts of the root and the development of normal tissues and rotten tissues were observed by light microscopy (Leica DM6000B; Leica Microsystems, IL, Germany).

*Huangqi* samples of 1, 2, 3, 4, and 5 years of age were used for microscopic examination of growth rings. Root segments were cut at approximately 2 cm from the root-head, excluding any rotten tissue. Parts of the segments were cut (5 mm thick), and from these, paraffin sections were prepared (as described previously) for light microscopic observation. Other parts of the segments were sectioned at 1-mm thickness by hand and stained with phloroglucinol-HCl. The growth rings in these sections were counted with the naked eye and also under a light microscope.

### Histochemical analysis

*Huangqi* samples were sectioned using a freezing microtome (Leica CM1850 UV, 30–40 μm) at 20–40-μm thickness, and the sections were stained with 5% sodium hydroxide solution. After few minutes, the sections were observed using fluorescence microscopy (Leica DM6000B; Leica Microsystems, Fluo, Germany) with a green filter (Leica Microsystems, Germany) and an emission wavelength of 420 nm. *Huangqi* samples were also sectioned using a freezing microtome (Leica CM1850 UV, 30–40 μm) at 20–40-μm thickness, and the sections were stained with 5% vanillin-sulfuric acid solution. After few minutes, saponins may become red or purple. Sections for a negative control experiment were treated with 70% alcohol for 1 month to remove flavonoids and saponins. These sections were then stained with 5% sodium hydroxide solution or 5% vanillin-sulfuric acid solution, as described previously.

### HPLC analysis

#### Preparation of different *Huangqi* tissues

*Huangqi* samples were classified into three categories based on the extent of rotten tissue: (A) “normal,” (B) “small rotten heart” (rotten tissue <1/3 of the total diameter), and (C) “large rotten heart” (rotten tissue ≥1/3 of the total diameter). Each category comprised three root samples. The normal and “small rotten heart” root samples were sliced (3 mm thick). The normal root slices were then divided into seven parts: periderm (Pd), secondary phloem (SPh) (outside to inside: SPh1 and SPh2), and secondary xylem (SX) (outside to inside: SX1, SX2, SX3, and SX4). The “small rotten heart” samples were partitioned using the same steps, but rotten heart (Ho) included the secondary xylem. The “large rotten heart” root samples were cut into six parts: Pd, SP (outside to inside: SPh1, SPh2), and SX (outside to inside: SX1, SX2) and Ho. All samples were crushed, dried at 60 °C, and sieved through a No. 4 mesh(diameter 250 ± 9.9 μm; Yu Ding Standard Screen Factory, Shaoxing, Zhejiang Province).

#### Preparation of test sample solutions

##### Flavonoids

In this study, we used the sample solution preparation method for *Huangqi* described in the Pharmacopoeia [1]. One gram of sample powder was reflux heated in 50 mL methanol for 4 h. The reduced weight was then complemented with methanol and the extract was well hand shaken and filtered. Twenty-five milliliters of the filtered fluid was recovered and dried. The residue was dissolved in methanol in a 5-mL volumetric flask and methanol was added to the scale line.

##### Saponins

Four grams of sample powder was soaked in 40 mL methanol in a Soxhlet extractor overnight, and then reflux heated for 4 h. The filtered fluid was recovered and dried. The residue was dissolved in 10 mL water. The samples were then extracted four times with 40 mL of water-saturated n-butyl alcohol. The n-butyl alcohol–water extracts were then pooled and washed four times with ammonia solution. The ammonia solution was discarded after each wash. The n-butyl alcohol fraction was dried. The residue was dissolved with methanol in a 5-mL volumetric flask and methanol was added to the scale line. Each sample preparation was performed in triplicate.

### HPLC conditions

The samples were analyzed using an Agilent 1260 HP LC system (Agilent, USA), equipped with DAD and ELSD (Agilent) detectors. Flavonoids and saponins were separated on a Kromasil C18 column (250 × 4.6 mm, 5 μm; Sweden). For flavonoids, the mobile phase consisted of acetonitrile (A) and 0.2% formic acid (B) 0–12 min, 20–37% A; 12–16 min, 37–40% A; 16–22 min, 40–50% A; 22–28 min, 50%–95 A; 28–32 min, 95–20% A; 32–42 min, 20% A. The sample injection volume was 20 μL, the flow rate was 1 mL/min, and the column temperature was 30 °C. The UV detection was set at 254 nm. For saponins, the mobile phase was acetonitrile:water (36:64), and the flow rate was 1 mL/min. For ELSD detection, the tube temperature was 100 °C, and the spray chamber temperature was 30 °C. The carrier gas was N_2_, the flow rate was 1.5 mL/min, and sample injection volume was 20 μL. The column temperature was 30 °C.

### Statistical analysis

The standard curves of the HPLC method, the correlation between growth rings and age, and correlations between the concentrations of the six examined bioactive constituents (calycosin, calycosin-7-glucoside, formononetin, ononin, astragaloside A, and astragaloside II) and age were analyzed using SPSS 19.0 software. The T test we used was two-sided. P value <0.05 indicated that there was significant difference, whereas P ≥ 0.05 indicated that there was no significant difference. Data of the concentration of six compounds are expressed as the mean ± standard deviations.

### Information of experimental design and resources

The information of experimental design, statistics, and resources used in this study are attached in Minimum standards of reporting checklist as Additional file 1.

## Results

### Anatomy of *huangqi*

**Fig. 1:**
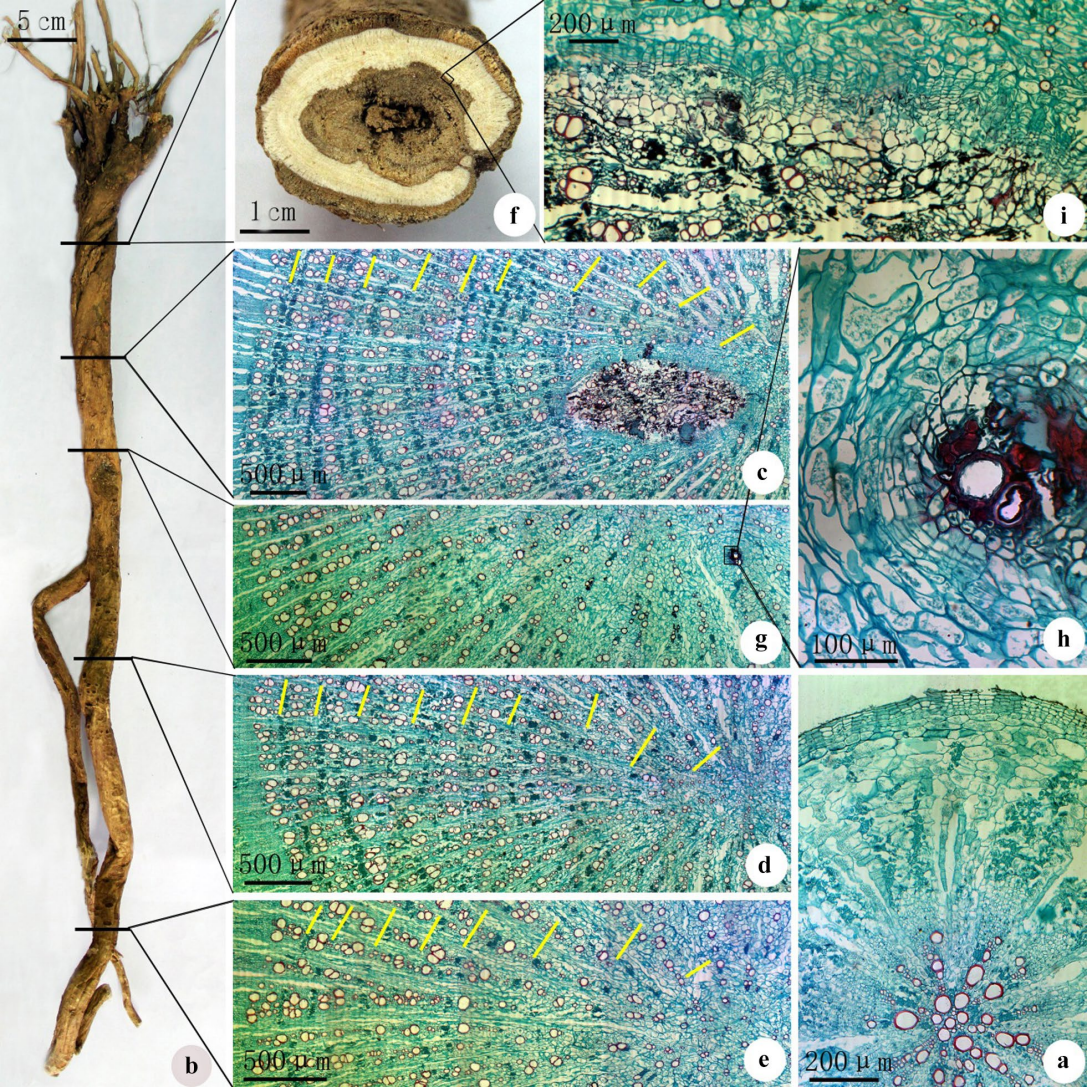
Pictures of the root anatomy of *Huangqi* from Hunyuan County, Shanxi Province. **a** Secondary structure of the root. **b** Macroscopic examination. **c** Rotting microstructure in the *upper portion* of the root. **d** *Middle portion* of the root. **e** *Lower portion* of the root. **f** Cross-section of rotten heart root. **g**, **h** Early development of the rotten heart at low and high magnifications. **i** Microscopic cross-sectional examination at the junction of healthy and rotten tissue. The *yellow arrows* represent growth rings

The typical secondary structure of *huangqi* from Shanxi Province consists primarily of a periderm and secondary vascular tissue (Fig. 1a). The periderm consisted of a cork layer, cork cambium, and phelloderm. The secondary vascular tissue was developed and included secondary xylem, vascular cambium, and secondary phloem. The secondary phloem consisted of many fiber bundles. The phloem rays were curved, with fissures close to the periderm. The vascular cambium consisted of five to seven layers of cells forming a circular line. The xylem rays consisted of two to three rows of cells and several bundle-like wood fibers were observed. The vessels were individually dispersed or gathered into small clusters of two to six vessels.

In the xylem, clusters of wide and narrow vessels were arranged alternatively as radial arrays. The clusters of wide vessels consisted of two to six large-diameter vessels, whereas numerous fiber bundles accompanied the clusters of narrow vessels. This alternating radial arrangement of wide and narrow vessels, together with the connection of vessels arranged in a tangential direction, forms very distinct growth rings. There were ten growth rings in the zone 20–40 cm from the root-head (Fig. 1b, c), nine in the zone 40–55 cm from the root-head, (Fig. 1b, d), and eight in the zone 55–70 cm from the root-head (Fig. 1b, e). Growth rings were evident from the root-head to the root-tail of *huangqi*, with the number decreasing toward the root-tail.

Rotten heart is primarily composed of decaying root tissue, which is similar to the structure of the periderm. In thick *huangqi,* a rotten heart is typically found in the area <30 cm from the root-head (Fig. 1b, f). The circle of parenchyma cells surrounding the clusters of vessels in the secondary xylem was transformed into cork cambium (Fig. 1g, h). The cork layer extended inward, causing necrosis in the secondary xylem. The growth rings were, however, still clearly visible. The cork cambium in the secondary xylem may became inactive. However, the peripheral parenchyma cell layer was converted to new cork cambium, producing inner cork layers. As this abnormal cambium production continued, the abnormal cork layer gradually increased in thickness. This portion of the secondary xylem gradually progressed from necrosis to decay (Fig. 1f, i).

### Relationship between growth rings and actual age in *Huangqi*

**Fig. 2:**
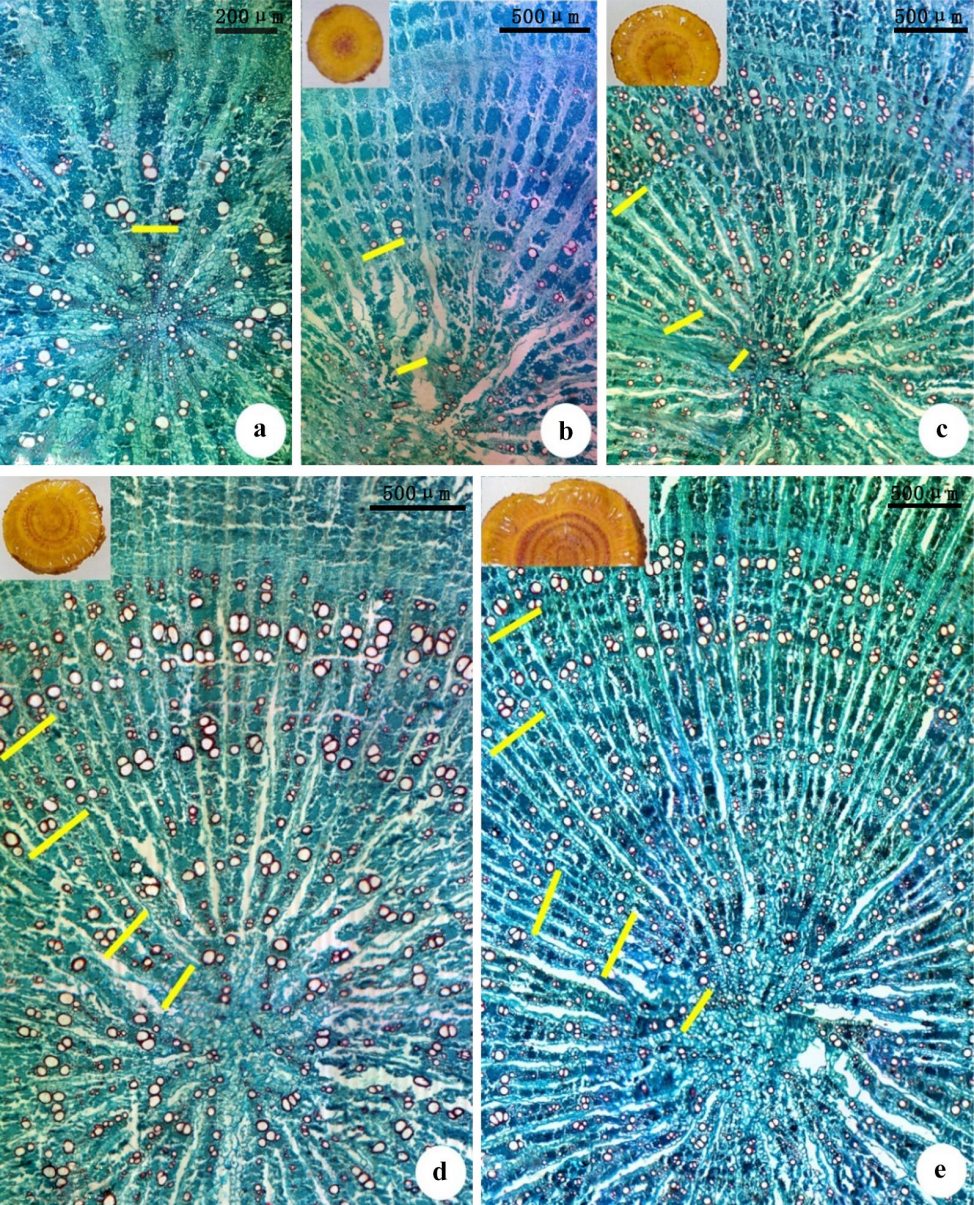
The growth rings in *huangqi* from Hunyuan County, Shanxi Province. **a** Growth rings in 1-year-old roots; **b** growth rings in 2-year-old roots; **c** growth rings in 3-year-old roots; **d** growth rings in 4-year-old roots; **e** growth rings in 5-year-old roots

After staining of the transverse sections with phloroglucinol-HCl, the growth rings became clearly visible red rings. The paraffin sections showed that the growth rings were composed of large-caliber vessels. These vessels were bound by clear layers of late wood (Fig. 2), More growth rings were identified by light microscopy than by unaided observation because the innermost growth ring was barely visible with the naked eye after phloroglucinol-HCl staining (Table 1). In *huangqi* roots of less than 5 years of age, the number of growth rings observed with the naked eye after staining with phloroglucinol-HCl plus 1 was equal to the actual number of growth rings. SPSS 19.0 software was used to analyze the correlation between growth rings (X) and age (Y). The result showed a significant correlation (Y = X, r = 1.000, P = 0.000).

### Validation of the HPLC method

Flavonoids and saponins were quantified by single-point calculation and two-point calculation (Additional file 2). The calibration curves of calycosin, calycosin-7-glucoside, formononetin, ononin, astragaloside A, and astragaloside II all showed good linearity, with significant correlation coefficient (Table 2). The limits of detection (LODs; S/N = 0.1) and limits of quantification (LOQs; S/N = 44) for flavonoids and saponins were <6 ng and 16 ng, respectively. The performance parameters evaluated were as follows: precision, recovery, repeatability, and stability at 0, 5, 10, 15, 20, 25, and 30 h. All relative standard deviations (RSDs) were <2%.

**Table 2:** The standard curves of four flavonoids and two saponins

Compound	Linear regression equation^a^	Correlation coefficient (r)	Linear range (μg)	P value
Calycosin	Y = 5273.7X − 0.7002	0.9999	0.0795–1.4310	0.0000
Calycosin-7-glucoside	Y = 3214.5X + 110.92	0.9996	0.1236–5.3560	0.0000
Formononetin	Y = 745.58X + 13.24	0.9999	0.0924–6.1600	0.0000
Ononin	Y = 4175.4X + 11.209	0.9999	0.0350–0.7000	0.0000
Astragaloside A	Y = 1.4106X + 2.3673	0.9995	0.3054–30.540	0.0000
AstragalosideII	Y = 1.5954X + 1.8899	0.9996	0.4240–16.9600	0.0000

^a^For flavonoids, Y is the peak area score of analyte, X is the concentration of analyte (µg/mL); For saponins, Y is the logarithm of peak area score, X is the logarithm of concentration

### Affect of age on the bioactive constituents in *Huangqi*

**Fig. 3:**
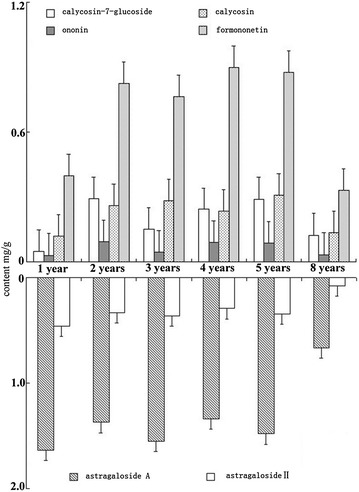
Influence of growth years on the concentrations of flavonoids and saponins in *Huangqi* from Hunyuan County, Shanxi Province. Sample analysis was conducted by HPLC using internal standards for quantification. Data are expressed as the mean ± standard deviations from three samples, each of which was determined in triplicate

*Huangqi* samples of different ages were analyzed by HPLC for the concentrations of four flavonoids and two saponins (Additional file 3). The correlations between years of growth and the four flavonoids were as follows: calycosin-7-glucoside (P = 0.88, r = 0.08), ononin (P = 0.848, r = −0.102), calycosin (P = 0.859, r = −0.095), and formononetin (P = 0.657, r = −0.233). There were thus no significant correlations between these four flavonoids and years of growth. Among these four flavonoids, formononetin was the most abundant and ononin the least for the sample ages examined (Fig. 3). Concentrations of the other two intermediate flavonoids were largely equivalent during this period. However, the amount of all flavonoids, with the exception of ononin, was two-fold higher during the 2nd to 5th year of growth than during the 1st and 8th years of growth. In contrast, the roots had similar saponins concentration during the first 5 years of growth, followed by proportional decreases in astragaloside A and astragaloside II concentrations in the 8th year of growth (Fig. 3). Astragaloside A was consistently the main saponin, with concentrations >threefold higher than astragaloside II. The SPSS analysis results for the relationship between years of growth and astragaloside A revealed a high correlation (P = 0.029, r = −0.909). The relationship between years of growth and astragaloside II also showed high correlation (P = 0.012, r = −0.858).

### Histochemical localization of flavonoids and saponins in *Huangqi*

**Fig. 4:**
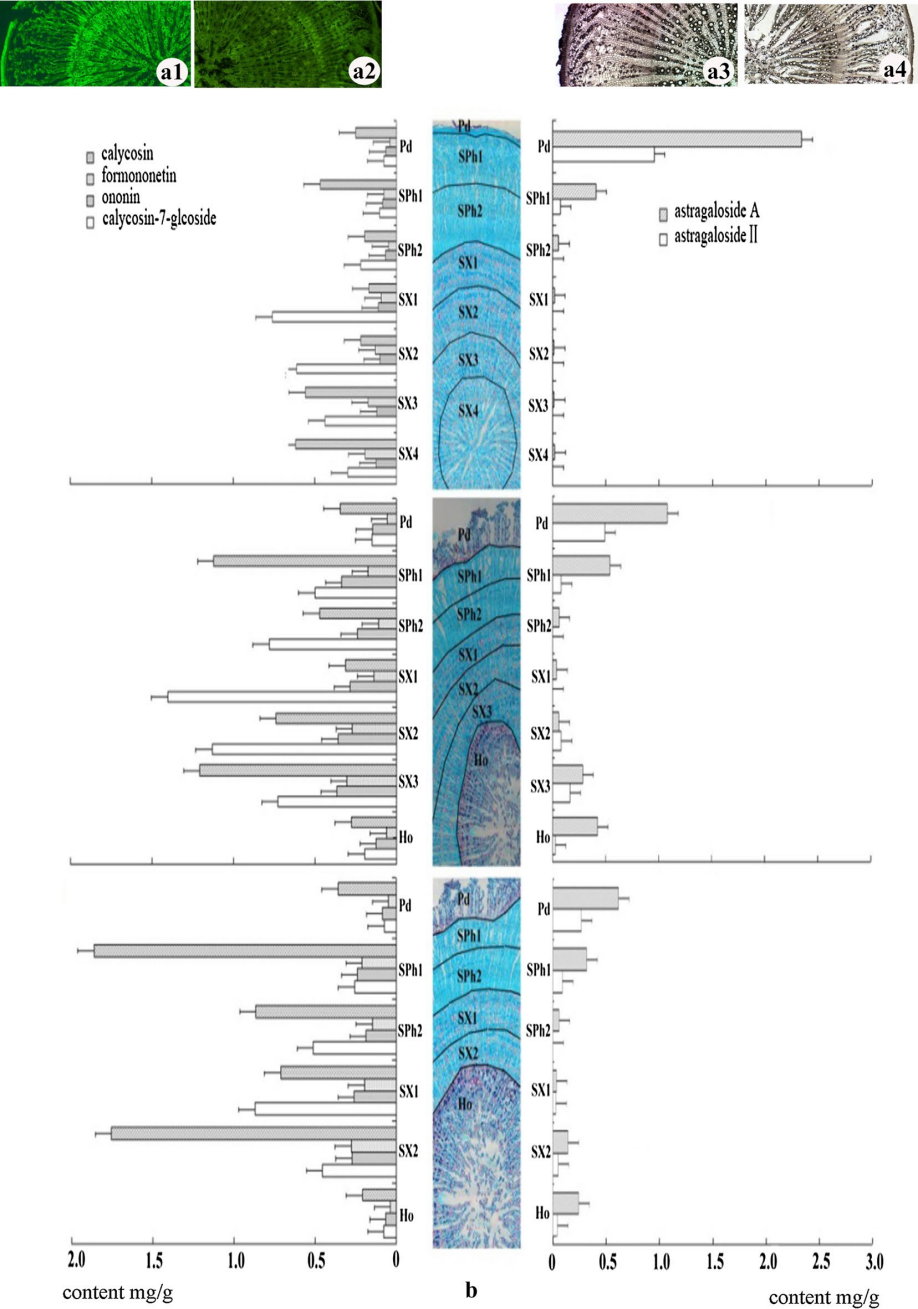
Quantitative localization of the main flavonoids and saponins in *huangqi*. (**a1**) Segment stained with 5% sodium hydroxide solution compared with the negative control segment (**a2**). (**a3**) Segment stained with 5% vanillin-sulfuric acid solution or unstained compared with the negative control segment (**a4**). **b** The concentration of four flavonoids and two saponins in different tissues. Data are expressed as the mean ± standard deviations from three samples, each of which was determined in triplicate. The slices were divided into seven areas, from the outside to inside, as follows: Pd, periderm; SPh1 and SPh2, secondary phloem zones 1–2; SX1, SX2, SX3, and SX4, secondary xylem zones 1–4; Ho, rotten heart

Flavonoids were concentrated in the secondary xylem to a greater extent than in the secondary phloem (Fig. 4A1) compared with the negative control segments (Fig. 4A2). In contrast, saponins were concentrated to a greater extent in the periderm and secondary phloem than in the secondary xylem of *huangqi* (Fig. 4A3). Negative control segments showed no staining of the periderm, secondary xylem, or secondary phloem (Fig. 4A4).

### HPLC analysis of flavonoids and saponins in the different tissues of *Huangqi*

The different tissue layers in *Huangqi* roots exhibited different compositions of flavonoids and saponins. In normal roots, the total concentration of the four flavonoids (ononin, calycosin, calycosin-7-glucoside, and formononetin) decreased toward the periderm (secondary xylem > secondary phloem > periderm) and increased toward the cambium (Fig. 4b). Ononin, calycosin, and calycosin-7-glucoside showed the same distribution pattern, with concentration decreasing toward the periderm (secondary xylem > secondary phloem > periderm). In contrast, formononetin concentration gradually increased from the periderm to the cambium, with the highest concentration in the secondary xylem adjacent to the cambium. In the secondary xylem, however, formononetin concentration gradually decreased toward the center of the root.

In the rotten heart roots, the distribution and total concentration of the four flavonoids were similar to those in the normal roots. However, in the rotten heart structure, the concentrations of the four flavonoids were proportionally lower than those in the surrounding normal tissue. In the periderm, flavonoid concentrations were not affected by the size of the rotten heart. In normal secondary xylem and secondary phloem, the total concentration of the four flavonoids in rotten heart root was significantly higher than that in normal roots. Calycosin-7-glucoside and ononin were not affected in the rotten heart structure; however, in normal secondary xylem and secondary phloem, calycosin and calycosin-7-glucoside concentrations increased with an increase in the extent of rotten heart.

In normal roots, the concentrations of astragaloside A and astragaloside II in the periderm were significantly higher than those in secondary xylem and secondary phloem. The total concentration of the two saponins was higher in rotten roots than in normal roots and their concentration distribution in different tissues was dependent on the size of the rotten heart. The rotten structures accumulated the two saponins (Additional file 3).

### Affect of rotten heart on flavonoids and saponins in 8-year-old *Huangqi*

**Fig. 5:**
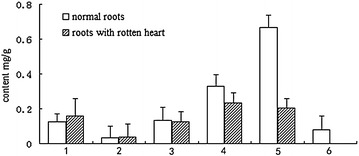
Influence of rotten heart on the total concentrations of flavonoids and saponins in 8-year-old *huangqi*. *1* calycosin-7-glucoside, *2* ononin, *3* calycosin, *4* formononetin, *5* astragaloside IV, *6* astragaloside II. Sample analysis was conducted by HPLC using internal standards for quantification. Data are expressed as the mean ± standard deviations from three samples, each of which was determined in triplicate

Total concentrations of the four flavonoids (ononin, calycosin, calycosin-7-glucoside, and formononetin) and two saponins (astragaloside A and astragaloside II) were determined by HPLC (Additional file 3). The flavonoids were not significantly affected by the development of a rotten heart (Fig. 5). In contrast, the concentration of astragaloside A in normal roots was approximately three times higher than that in rotten roots, whereas astragaloside II was not detected in rotten roots. The saponin concentrations of *huangqi* are thus selectively decreased if a rotten heart develops.

## Discussion

### Identification of the age of *Huangqi*

The number of years of growth and the growth region both affect the quality of *huangqi*. Hunyuan County (Shanxi Province) is the main production region of *Dao*-*di huangqi,* which has a long lifespan. One growth ring includes one ring of larger vessels and one ring of narrower vessels. The larger vessels develop in the growing seasons (spring and summer), whereas the narrower vessels develop later (autumn). The growth rings in *huangqi* roots are located in the xylem [22, 23]. *Huangqi* from Hunyuan County has clearly visible growth rings, and the number of growth rings correspond to the plants’ real age. For *huangqi* that has been cultivated for less than 5 years, a very simple and convenient method for determining the number of years of growth is staining of hand-sliced sections with phloroglucinol-HCl, and observing with the naked eye.

### Tissue-specific flavonoid and saponin profiles in *Huangqi*

The concentration of two saponins (astragaloside A and astragaloside II) decreases toward the center in normal *huangqi*, whereas the concentration of four flavonoids (ononin, calycosin, calycosin-7-glucoside, and formononetin) showed the opposite trend. These results are consistent with the findings of a previous study [16]. This contrasting trend in concentration distribution between flavonoids and saponins in different tissues could be a general characteristic of *huangqi*, regardless of the growth region or year of growth.

### Rotten heart disrupts flavonoids and saponins in *Huangqi*

In old huangqi plants, there is a high likelihood that the root-head will be hollow. The development of a rotten heart does not affect the distribution and accumulation of the four main flavonoids and two main saponins in normal tissue. However, in rotten heart, the concentrations of flavonoids and saponins were distinct from those in the surrounding normal secondary xylem. The concentrations of the four flavonoids in the rotten heart were similar to those in the periderm, but significantly lower than those in the secondary xylem and secondary phloem. As the rotten heart becomes larger, the distribution of flavonoids and saponins changed.

The rotten heart in *huangqi* is typically removed before the medicinal compounds are extracted, which increases the concentration of flavonoids but decreases the concentration of saponins. Thus, the pharmacological activities of *huangqi* may vary depending on the extent of rotten heart, as a consequence of changes in the concentrations of flavonoids and saponins.

## Conclusion

The growth year of *huangqi* (Hunyuan County, Shanxi Province) could be determined very simply and conveniently by naked eye. The content of total flavonoids and saponins in *huangqi* reached the maximum at 5-year-old. The rotten heart in *huangqi* influenced the distribution and concentration of bioactive constituents.

## Additional files

**Additional file 1.** Minimum standards of reporting checklist.

**Additional file 2.** Raw data in Table 2.

**Additional file 3.** The raw data in Figs. 3, 4 and 5.

## Abbreviations

Pd: periderm
SPh: secondary phloem
SX: secondary xylem
Ho: hollow structure
